# Tricyclic flavonoids with 1,3-dithiolium substructure

**DOI:** 10.3762/bjoc.8.226

**Published:** 2012-11-16

**Authors:** Lucian G Bahrin, Peter G Jones, Henning Hopf

**Affiliations:** 1 Department of Chemistry, “Al. I. Cuza” University of Iasi, 11 Carol I Bv., RO-700506 Iasi, Romania; 2 Institute of Organic Chemistry, Technical University of Braunschweig, Hagenring 30, D-38106 Braunschweig, Germany; 3 Institute of Inorganic and Analytical Chemistry, Technical University of Braunschweig, Hagenring 30, D-38106 Braunschweig, Germany

**Keywords:** aminals, benzopyrans, dithiocarbamates, dithiolium salts, flavonoids

## Abstract

The synthesis of new 3-dithiocarbamic flavonoids has been accomplished by the reaction of the corresponding 2-hydroxyaryl dithiocarbamates with aminals. These flavonoids were obtained as a mixture of diastereoisomers, the *anti* isomer being the major one. The heterocyclization of these compounds provided novel tricyclic flavonoids bearing a 1,3-dithiolium-2-yl ring fused at the 3,4-carbon positions of the benzopyran moiety.

## Introduction

The great diversity in physical, chemical and biochemical properties of flavonoids gives them the ability to influence the biological activity of plants, microbes and animals [1–3]. Flavonoids are known to be good antioxidants and this is believed to be the most probable mechanism of protection that these compounds offer against conditions such as cancer or cardiovascular disease [4].

**Figure 1:**
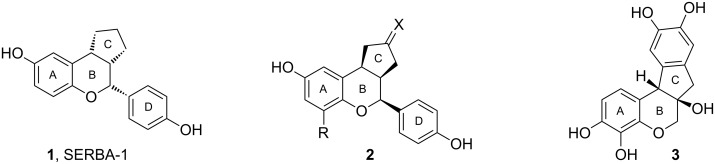
Polycyclic flavonoids.

SERBAs, or selective estrogen receptor β agonists, are benzopyran derivatives that interact with estrogen receptor subtypes α and β. The interest in this type of compound led to the synthesis of derivative **1**, a selective estrogen receptor β agonist (SERBA-1, Figure 1) [5]. Studies that focus on the structure–activity relationship were reported [6–7]. It was shown that a cyclopentane ring at the 3,4-carbon positions (labeled C, Figure 1) leads to a substantial rise in the binding affinity for estrogen receptor β. Further improvements in binding selectivity were obtained by combining the modifications performed on the C ring with modifications performed on the A ring, e.g. **2**, Figure 1 [8].

Extracts from *Hematoxylum campechianum* and *Caesalpinia sappan* are known to be effective in the treatment of various conditions, such as diarrhea, dysentery, dyspepsia, leucorrhea, and diabetic complications [9–10]. The main components of *H. campechianum* and *C. sappan* are hematoxylin (**3**, Figure 1) and brazilin; the basic structures of the two contain a 3,4-cycloalkyl-fused benzopyran unit. In addition to this, they are known to possess anti-inflammatory activity, inhibit the lens-aldose reductase, and decrease the blood glucose level [11–12]. Moreover, compound **3** was shown to inhibit HIV-1 integrase [13–14].

Several molybdenum and tungsten oxobisdithiolene complexes have been synthesized from a tricyclic flavonoid bearing a 1,3-dithiolium-2-yl unit as **C** ring [15]. These complexes have been electrochemically investigated to provide a better understanding of enzymatic activities in thermophilic and hyperthermophilic organisms.

This paper outlines the synthesis of novel tricyclic flavonoids bearing a 1,3-dithiolium-2-yl ring fused at the 3,4-carbon positions of the benzopyran moiety, utilizing the heterocyclization of corresponding 3-substituted dithiocarbamic flavonones.

## Results and Discussion

**Scheme 1:**
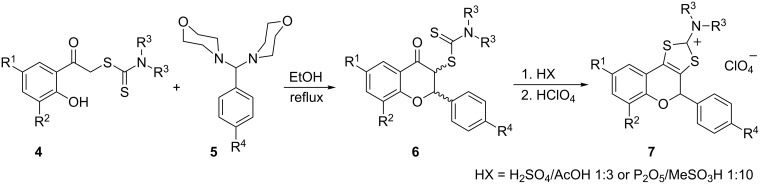
The synthesis of flavonoids **6** and **7**.

The treatment of several dithiocarbamates of type **4** with aminals has been reported to provide new substituted flavanones and related 4-chromanones [16]. Based on this study, we decided to investigate the synthesis of new flavonoids bearing a 1,3-dithiolium-2-yl ring fused at the 3,4-carbon positions of the benzopyran core. The reaction of dithiocarbamates **4** with aminals **5** provided 3-substituted dithiocarbamic flavanones **6** as a mixture of diastereoisomers (Scheme 1, Table 1). The analytical and spectroscopic data are in agreement with the structures. 2-Hydroxyphenacyl dithiocarbamates **4** are known compounds and were prepared following the reported experimental procedures [16–19]. Aminals **5** were synthesized according to the literature procedures [20–21].

**Table 1:** Flavonoids **6** and **7**.

**6**, **7**^a^	R^1^	R^2^	R^3^–N–R^3^	R^4^	Yields for **6**, (%)	Yields for **7**, (%)

**a**	H	H	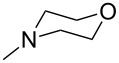	H	88	73
**b**	H	H	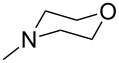	OMe	83	60
**c**	H	H	NEt_2_	OMe	60	76
**d**	Br	H	NEt_2_	Cl	71	71
**e**	Br	H	NEt_2_	H	81	64
**f**	Br	Br	NEt_2_	H	62	80
**g**	I	I	NEt_2_	H	64	80
**h**	I	I	NEt_2_	Cl	87	65

^a^**7g** and **7h** prepared in the presence of P_2_O_5_/MeSO_3_H (1:10) mixture.

**Figure 2:**
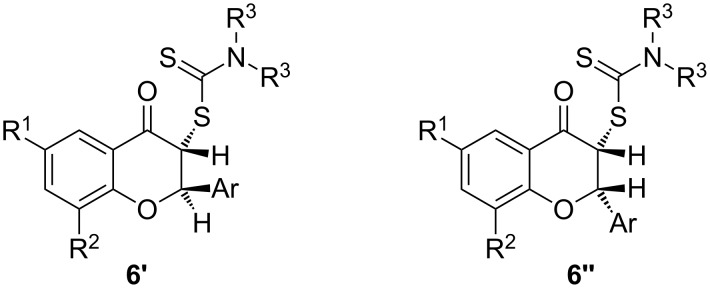
Diastereoisomers of flavonoids **6**.

Although the original short synthetic communication on flavonoids of type **6** [16] did not mention the existence of diastereoisomers, we always identified an inseparable mixture of two diastereoisomers. Each isomer could be identified, however, by their different NMR chemical shifts and coupling constants of the H-2 and H-3 hydrogen atoms (**6′** and **6″**, Figure 2). Furthermore, a major isomer was identified for all flavonoids **6a**–**i**. We therefore decided to investigate the structures of the two diastereoisomers, together with the influence of substituents on their ratio. In principle, the two isomers could have the hydrogen atoms at the 2 and 3 positions directed either to opposite sides or to the same side of the benzopyrane ring. The relative orientation of the two hydrogen atoms would, of course, be expected to have an influence on the magnitude of their coupling constants. Moreover, it is reasonable to assume that the most stable isomer is that with an *anti* orientation of the two hydrogen atoms (e.g., **6′**, Figure 2).

The coupling constants and diastereoisomeric ratios of flavonoids **6** are presented in Table 2. Data analysis indicates that the highest diastereoisomeric ratios are obtained for **6a** and **6b**, i.e., the flavonoids with an *N*-morpholinyl carbodithioate substituent. Although no significant influence of the substituents on ^3^*J*_H2-H3_ coupling constants of *syn* isomers was recorded, an unexpected variation of the coupling constant with the R^2^ substituent was recorded for the *anti* isomers. The replacement of the hydrogen atom at C8 by a bulky bromine or iodine atom induces a decrease of the ^3^*J*_H2-H3_ coupling constant by about 2 Hz (**6f**–**h**).

**Table 2:** Coupling constants and diastereoisomers ratio of flavonoids **6**.

**6**	**a**	**b**	**c**	**d**	**e**	**f**	**g**	**h**

^3^*J*_H2-H3_ *anti* (Hz)	9.3	9.8	9.3	9.5	9.5	7.6	7.2	7.8
^3^*J*_H2-H3_* syn* (Hz)	4.0	4.3	4.3	4.0	4.2	4.0	4.1	3.8
*anti*/*syn* ratio	86:14	77:23	73:27	72:28	69:31	65:35	64:36	69:31

**Figure 3:**
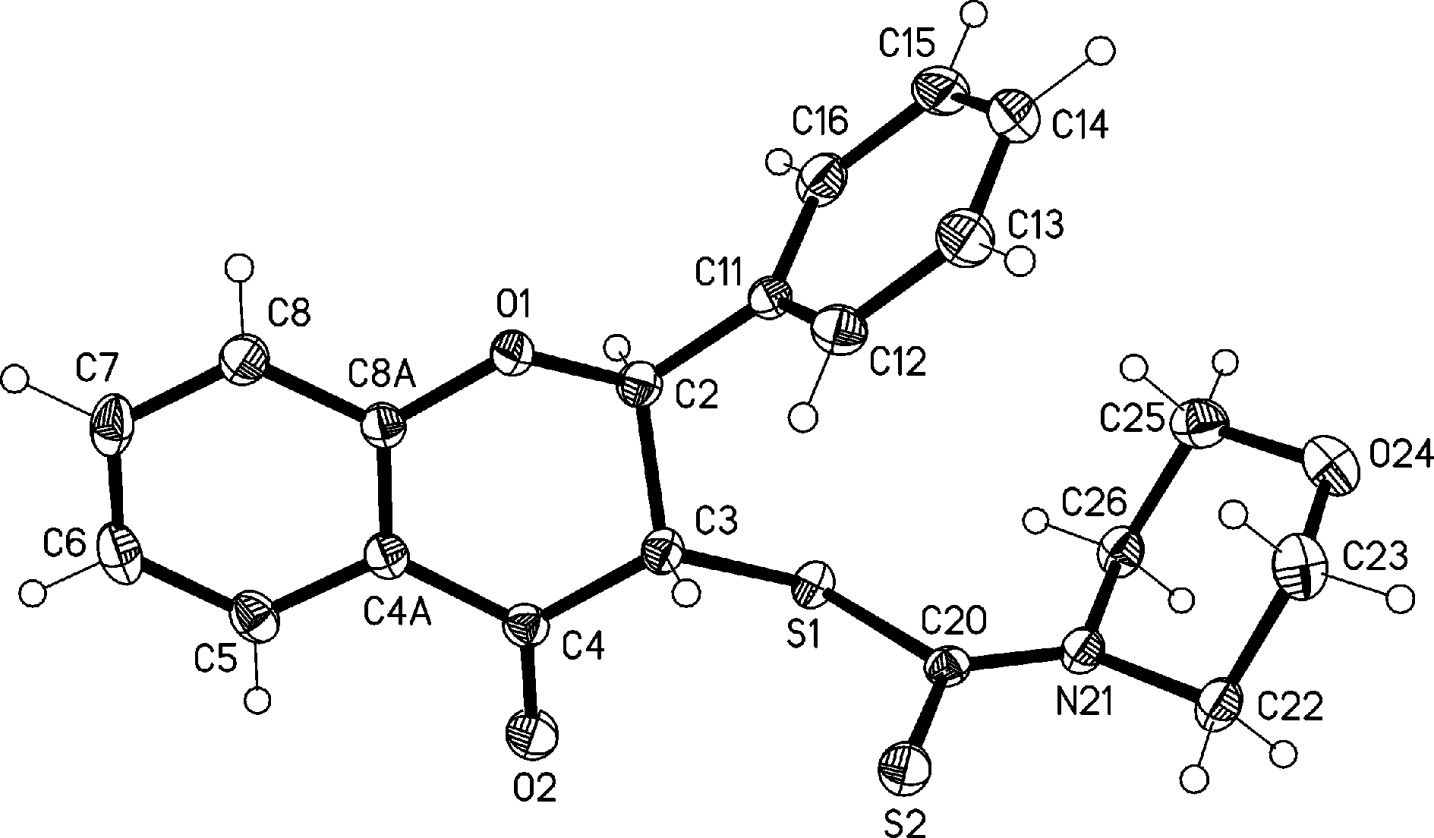
Molecular structure of flavonoid **6a** in the solid state. Ellipsoids represent 50% probability levels. Torsion angle C(11)–C(2)–C(3)–S(1): −59.19(14)°.

**Figure 4:**
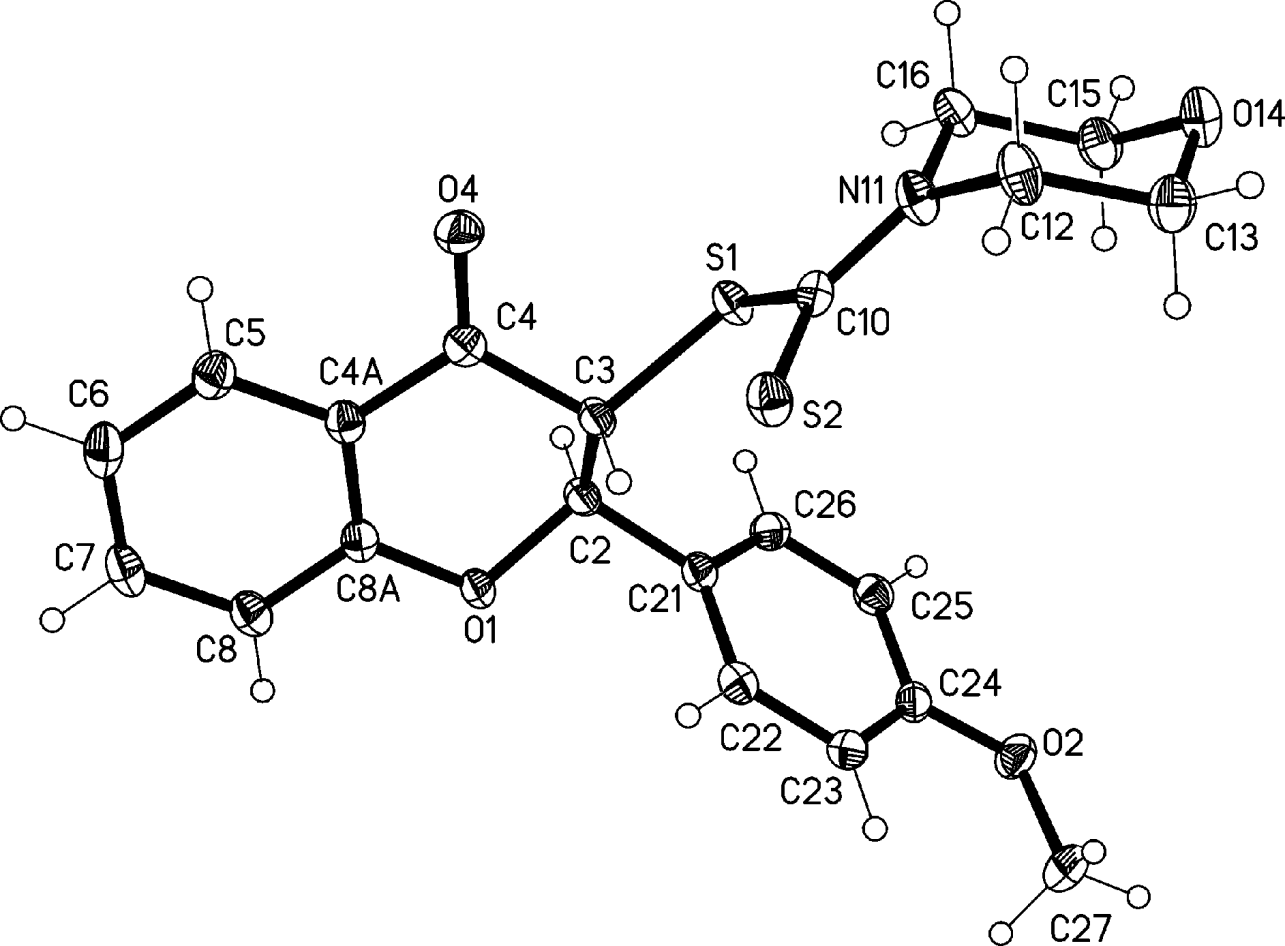
Molecular structure of flavonoid **6b** in the solid state. Ellipsoids represent 50% probability levels. Torsion angle C(21)–C(2)–C(3)–S(1): 59.72(11)°.

The structural information provided by the NMR data has been unambiguously corroborated by X-ray analysis. The structures of the *anti* isomers of flavonoids **6a** and **6b** are presented in Figure 3 and Figure 4, respectively.

The main synthetic approach for various substituted 1,3-dithiolium-2-yl cations consists of the cyclization of phenacyl carbodithioates by using acid as catalyst. The heterocyclization of compounds **6** should, hence, provide new tricyclic fused flavonoids **7** by using an appropriate mixture of acids, as described above (Scheme 1, Table 1). Using a concentrated sulfuric acid/glacial acetic acid (1:3 v/v) mixture [22–24] the cyclization of dithiocarbamates **6a–f** takes place under mild reaction conditions. By heating the reaction mixture at 80 °C a homogeneous solution was obtained, which contains the corresponding 1,3-dithiolium ions. The addition of 70% perchloric acid to the cooled solution, followed by water yielded perchlorates **7a–f**, isolated as white crystalline products in good to excellent yields (60–80%). Attempts to cyclize *N*,*N*-dialkyldithiocarbamates **6g** and **6h** by using the above-mentioned or other common cyclization agents, according to the literature [25–27], led to degradation of the substrates, often accompanied by loss of molecular iodine. A literature survey revealed that a P_2_O_5_/CH_3_SO_3_H (1:10, m/v) mixture may be the cyclization agent of choice to obtain 1,3-dithiolium salts **7g** and **7h** as pure compounds and in high yields [28–29]. Thus, a suspension of **6g** or **6h** in three parts of the ‘‘superacid’’ mixture was stirred at room temperature for 30 min to give a solution that contained the corresponding 1,3-dithiolium cation. Addition of 70% perchloric acid and methyl acetate to this solution yielded perchlorates **7g** and **7h** as white crystalline products in 65–80% isolated yield.

**Figure 5:**
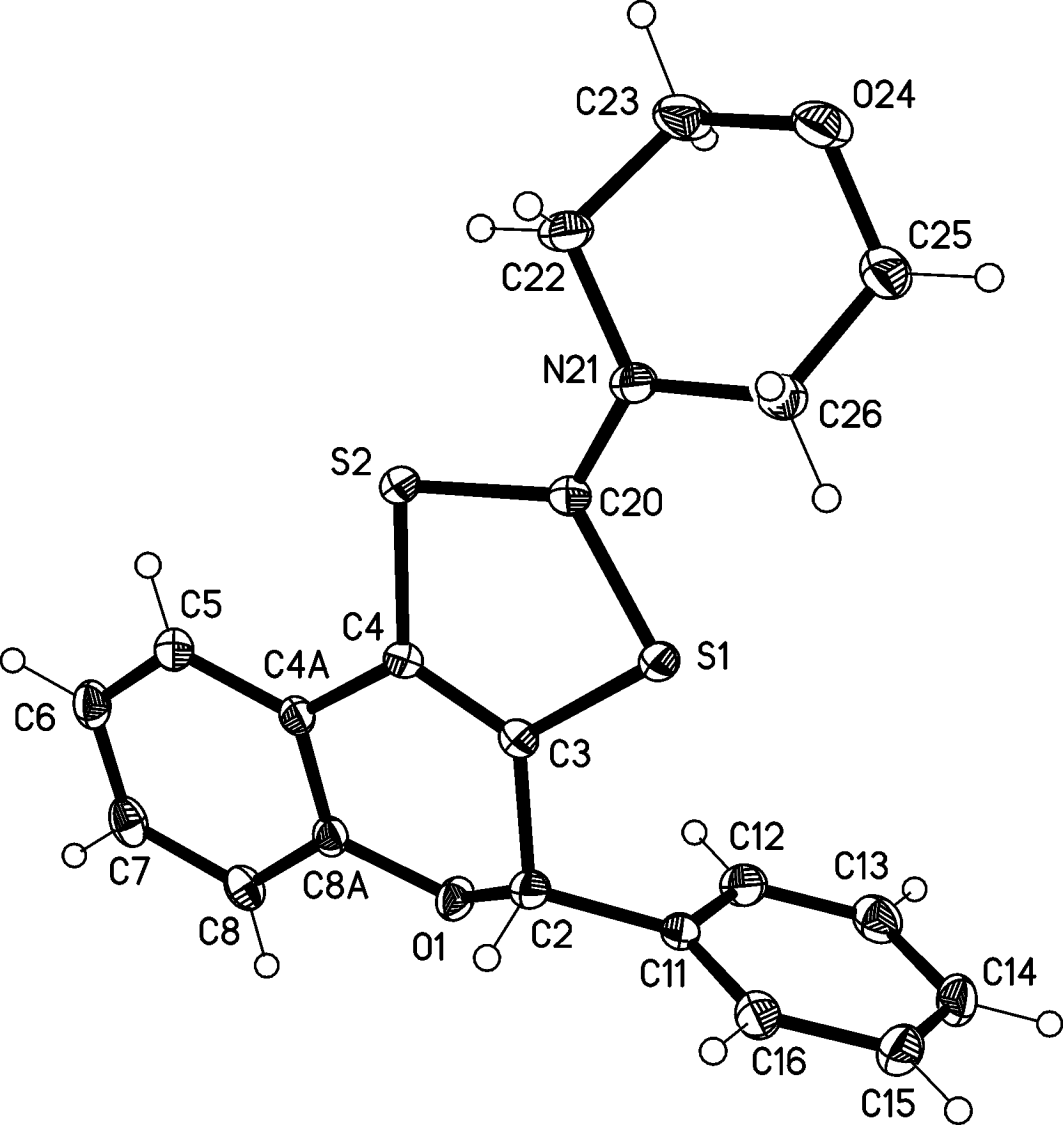
Molecular structure of flavonoid **7a** in the solid state. Ellipsoids represent 50% probability levels.

The structure of these charged tricyclic fused flavonoids was unambiguously proved by X-ray analysis. The structure of tricyclic flavonoid cation **7a** is presented in Figure 5. Structures of the perchlorate salts proved to be disordered, so the tetraphenylborate anion was used instead. The bond length C20–N21, 1.311(2) Å, corresponds to a double bond and thus to a formal positive charge at the nitrogen atom. Similarly, C3–C4, at 1.342(2) Å, is also a (marginally lengthened) double bond. The C–S bond lengths in the five-membered ring are approximately equal [1.727(1) – 1.746(1) Å].

The new 1,3-dithiolium cations are particularly prone to nucleophilic attack at their 2-positions [30]. This behavior opens the way for the synthesis of various substituted 1,3-dithiolic rings, especially those with a 2-ylidene moiety resulting from a *C*-nucleophile attack. Furthermore, by using oxygen, sulfur, phosphorus, or nitrogen nucleophiles, interesting and novel structures can in principle be obtained [31]. The above combination of the chemistry of 1,3-dithiol-2-yl cations and the biological properties of flavonoids may provide new compounds with interesting properties.

## Conclusion

The synthesis of 3-dithiocarbamic flavonoids has been accomplished by the reaction of the corresponding 2-hydroxyphenacyl dithiocarbamates with aminals. Under strongly acidic conditions these compounds provide novel tricyclic, fused flavonoids with a 1,3-dithiol-2-yl substructure. The reactivity of the latter derivatives could open the way for the synthesis of new flavonoids with potential biological activities.

## Supporting Information

File 1 Experimental and X-ray spectral data.
