# Supplementary material for: Tricyclic flavonoids with 1,3-dithiolium substructure
Source: Beilstein J Org Chem. 2012 Nov 16;8:1999–2003. doi: 10.3762/bjoc.8.226 (PMC3511035; doi:10.3762/bjoc.8.226)
Supplement: File 1 — Experimental and X-ray spectral data. [file Beilstein_J_Org_Chem-08-1999-s001.pdf]

Supporting Information

for

Tricyclic flavonoids with 1,3-dithiolium substructure

Lucian G. Bahrin*[1,2], Peter G. Jones[3] and Henning Hopf[2]

Address: [1]Department of Chemistry, "Al. I. Cuza" University of Iasi, 11 Carol I Bv., RO-700506 Iasi, Romania, [2]Institute of Organic Chemistry, Technical University of Braunschweig, Hagenring 30, D-38106 Braunschweig, Germany and [3]Institute of Inorganic and Analytical Chemistry, Technical University of Braunschweig, Hagenring 30, D-38106 Braunschweig, Germany

Email: Lucian G. Bahrin* - l.bahrin@tu-braunschweig.de

* Corresponding author

Experimental and X-ray spectral data

Experimental

General Remarks: Melting points: Büchi 510, uncorrected. IR: Bruker Tensor 27. ^{1}H and ^{13}C NMR: Bruker DRX 400 in $CDCl_3$ or DMSO-d_6 with TMS as internal standard at room temp. Chemical shifts are reported in ppm downfield from tetramethylsilane.

NMR data are presented for the major isomer (*anti*). MS: Finnigan MAT 90X, electron impact (EI). Elemental analyses: CE440 Elemental Analyser. All reagents were commercially available and used without further purification. 2-Hydroxyphenacyl dithiocarbamates **4** are known compounds and were prepared following the reported experimental procedures [16-29]. Aminals **5** were synthesized according to the literature procedures [20,21].

Flavonone 6a; **General procedure**: To a solution of 1-(2-hydroxyphenyl)-1-oxoethan-2-yl-morpholine-4-carbodithioate **4** (0.85 g, 3 mmol) in EtOH (30 mL) aminal **5** (R^4 = H, 0.79 g, 3 mmol) was added and the reaction mixture was heated under reflux for 2 h. After cooling the solid material was filtered off and purified by recrystallization from ethanol to give **6a** (1.02 g, 88%) as colorless crystals. Mp 147–148 °C; IR 2884, 1683, 1604, 1462, 1420, 1294, 1263, 1222, 1103, 975, 763, 700, 626 cm^{-1}; ^1H NMR ($CDCl_3$) δ 3.66 (m, 4H), 4.04 (m, 4H), 5.80 (d, *J* = 9.3 Hz, 1H), 5.90 (d, *J* = 9.3 Hz, 1H), 7.10 (m, 2H), 7.35 (m, 3H), 7.50 (m, 3H), 7.94 (m, 1H) ppm; ^{13}C NMR ($CDCl_3$) δ 58.3, 59.6, 66.2, 82.7, 118.2, 121.0, 122.1, 127.8, 128.6, 129.0, 129.6, 136.8, 160.6, 187.6, 193.7 ppm; EIMS *m/z*: 385.1 ($[M]^+$), 255.0 ($[M-C_5H_8NOS]^+$), 223.1 ($[M-C_5H_8NOS_2]^+$), 162.0 ($[C_5H_8NOS_2]^+$), 130.0 ($[C_5H_8NOS]^+$); Analysis calcd for $C_{20}H_{19}NO_3S_2$: C, 62.31; H, 4.97; N, 3.63; found: C, 62.53; H, 4.81; N, 3.86.

Flavonone 6b: Yield 83% (1.03 g); mp 125–126 °C; IR 3065, 2884, 1700, 1588, 1491, 1444, 1417, 1265, 1198, 1086, 826, 642, 539 cm^{-1}; ^1H NMR ($CDCl_3$) δ 3.80 (m, 4H), 4.11 (m, 4H), 5.74 (d, *J* = 9.8 Hz, 1H), 5.93 (d, *J* = 9.8 Hz, 1H), 6.89 (d, *J* = 9.8 Hz, 2H), 7.05 (m, 2H), 7.43 (d, *J* = 9.8 Hz, 2H), 7.53 (m, 1H), 7.94 (m, 1H) ppm; ^{13}C

NMR ($CDCl_3$) δ 55.3, 59.7, 66.1, 82.3, 113.8, 118.1, 120.9, 121.9, 127.7, 128.6, 129.1, 136.5, 159.9, 160.6, 187.8, 193.9 ppm; EIMS *m/z*: 415.1 ($[M]^+$), 285.1 ($[M-C_5H_8NOS]^+$), 253.1 ($[M-C_5H_8NOS_2]^+$), 162.0 ($[C_5H_8NOS_2]^+$), 130.0 ($[C_5H_8NOS]^+$); Analysis calcd for $C_{21}H_{21}NO_4S_2$: C, 60.70; H, 5.09; N, 3.37; found: C, 60.93; H, 4.91; N, 3.52.

Flavonone 6c: Yield 60% (0.72 g); mp 111–112 °C; IR 2875, 1682, 1599, 1460, 1418, 1292, 1219, 1179, 969, 822, 766, 607 cm^{-1}; ^1H NMR ($CDCl_3$) δ 1.19 (t, *J* = 7.1 Hz, 6H), 3.65 (q, *J* = 7.1 Hz, 2H), 3.79 (s, 3H), 3.96 (q, *J* = 7.1 Hz, 2H), 5.73 (d, *J* = 9.3 Hz, 1H), 5.92 (d, *J* = 9.3 Hz, 1H), 6.87 (d, *J* = 9.7 Hz, 2H), 7.05 (m, 2H), 7.44 (d, *J* = 9.7 Hz, 2H), 7.52 (m, 1H), 7.93 (m, 1H) ppm; ^{13}C NMR ($CDCl_3$) δ 11.5, 12.6, 47.3, 50.6, 55.4, 60.2, 82.7, 113.8, 118.2, 121.0, 121.9, 127.8, 128.6, 129.2, 136.5, 159.8, 160.7, 188.1, 192.0 ppm; EIMS *m/z*: 401.1 ($[M]^+$), 285.1 ($[M-C_5H_{10}NS]^+$), 253.1 ($[M-C_5H_{10}NS_2]^+$), 148.0 ($[C_5H_{10}NS_2]^+$), 116.0 $[C_5H_{10}NS]^+$); Analysis calcd for $C_{21}H_{23}NO_3S_2$: C, 62.81; H, 5.77; N, 3.49; found: C, 63.05; H, 5.91; N, 3.77.

Flavonone 6d: Yield 71% (1.03 g); mp 125–126 °C; IR 2983, 2700, 1681, 1596, 1492, 1461, 1267, 986, 821, 632 cm^{-1}; ^1H NMR ($CDCl_3$) δ 1.20 (t, *J* = 7.1 Hz, 6H), 3.65 (q, *J* = 7.1 Hz, 2H), 3.92 (m, 2H), 5.78 (d, *J* = 9.5 Hz, 1H), 5.82 (d, *J* = 9.5 Hz, 1H), 6.97 (d, *J* = 8.8 Hz, 1H), 7.32 (d, *J* = 8.6 Hz, 2H), 7.44 (d, *J* = 8.6 Hz, 2H), 7.60 (dd, *J* = 2.5, 8.8 Hz, 1H), 8.02 (d, *J* = 2.5 Hz, 1H) ppm; ^{13}C NMR ($CDCl_3$) 11.4, 12.6, 47.4, 50.7, 59.6, 82.4, 114.7, 120.2, 128.6, 128.7, 129.2, 130.2, 134.7, 134.8, 139.2, 159.3, 186.6, 191.1 ppm; EIMS *m/z*: 485.0 ($[M]^+$), 368.9 ($[M-C_5H_{10}NS]^+$), 337.0 ($[M-C_5H_{10}NS_2]^+$), 148.0 ($[C_5H_{10}NS_2]^+$), 116.0 ($[C_5H_{10}NS]^+$); Analysis calcd for $C_{20}H_{19}BrClNO_2S_2$: C, 49.54; H, 3.95; N, 2.89; found: C, 49.83; H, 4.09; N, 3.05.

Flavonone 6e: Yield 81% (1.09 g); mp 142–143 °C; IR 3080, 2770, 1686, 1594, 1465, 1412, 1265, 1200, 973, 824, 764, 660 cm^{-1}; ^{1}H NMR ($CDCl_3$) δ 1.16 (t, J = 7 Hz, 3H), 1,19 (t, J = 7 Hz, 3H), 3.64 (q, J = 7 Hz, 2H), 3.92 (q, J = 7 Hz, 2H), 5.81 (d, J = 9.5 Hz, 1H), 5.86 (d, J = 9.5 Hz, 1H), 6.98 (d, J = 7.8 Hz, 1H), 7.33 (m, 3H), 7.47 (m, 2H), 7.60 (dd, J = 2.5, 7.8 Hz, 1H), 8.01 (d, J = 2.5 Hz, 1H) ppm; ^{13}C NMR ($CDCl_3$) δ 11.5, 12.3, 47.3, 50.6, 59.5, 83.0, 114.5, 120.3, 122.4, 127.7, 128.6, 129.0, 130.1, 136.2, 139.2, 159.4, 186.8, 191.4 ppm; EIMS *m/z*: 449.0 ($[M]^+$), 333.0 ($[M-C_5H_{10}NS]^+$), 301.0 ($[M-C_5H_{10}NS_2]^+$), 148.0 ($[C_5H_{10}NS_2]^+$), 116.0 ($[C_5H_{10}NS]^+$); Analysis calcd for $C_{20}H_{20}BrNO_2S_2$: C, 53.33; H, 4.48; N, 3.11; found: C, 53.59; H, 4.62; N, 3.36.

Flavonone 6f: Yield 62% (1.04 g); mp 153–154 °C; IR 3080, 2977, 2770, 1686, 1594, 1465, 1412, 1265, 1201, 973, 824, 764, 660 cm^{-1}; ^{1}H NMR ($CDCl_3$) δ 1.22 (t, J = 7.3 Hz, 6H), 3.67 (q, J = 7.3 Hz, 2H), 3.95 (m, 2H), 5.77 (d, J = 7.6 Hz, 1H), 5.96 (d, J = 7.6 Hz, 1H), 7.35 (m, 5H), 7.88 (d, J = 2.3 Hz, 1H), 7.96 (d, J = 2.3 Hz, 1H) ppm; ^{13}C NMR ($CDCl_3$) δ 11.4, 12.7, 47.4, 50.7, 58.6, 82.7, 113.1, 114.6, 123.1, 128.4, 128.7, 128.8, 129.5, 134.3, 141.6, 155.9, 185.9, 191.0 ppm; EIMS *m/z*: 528.9 ($[M]^+$), 444.9 ($[M-C_5H_{10}NS]^+$), 412.9 ($[M-C_5H_{10}NS_2]^+$), 148.0 ($[C_5H_{10}NS_2]^+$), 116.0 ($[C_5H_{10}NS]^+$); Analysis calcd for $C_{20}H_{19}Br_2NO_2S_2$: C, 45.38; H, 3.62; N, 2.65; found: C, 45.61; H, 3.75; N, 2.99.

Flavonone 6g: Yield 64% (1.19 g); mp 152–153 °C; IR 3075, 1684, 1569, 1492, 1419, 1265, 1254, 1202, 973, 825, 760, 661 cm^{-1}; ^{1}H NMR ($CDCl_3$) δ 1.20 (t, J = 7.1 Hz, 3H), 1.24 (t, J = 7.1 Hz, 3H), 3.67 (q, J = 7.1 Hz, 2H), 3.95 (m, 2H), 5.82 (d, J = 7.2 Hz, 1H), 5.86 (d, J = 7.2 Hz, 1H), 7.16–7.54 (m, 5H), 8.12 (d, J = 2.1 Hz, 1H),

8.25 (d, J = 2.1 Hz, 1H) ppm; ^{13}C NMR ($CDCl_3$) δ 11.5, 12.7, 47.4, 50.6, 58.0, 83.1, 84.8, 87.5, 122.9, 127.3, 128.5, 129.0, 134.8, 136.4, 152.7, 158.7, 186.1, 191.0 ppm; EIMS *m/z*: 622.9 ($[M]^+$), 506.9 ($[M-C_5H_{10}NS]^+$), 474.9 ($[M-C_5H_{10}NS_2]^+$), 148.0 ($[C_5H_{10}NS_2]^+$), 116.0 ($[C_5H_{10}NS]^+$); Analysis calcd for $C_{20}H_{19}I_2NO_2S_2$: C, 38.54; H, 3.07; N, 2.25; found: C, 38.74; H, 3.25; N, 2.44.

Flavonone 6h: Yield 87% (1.71 g); mp 160–161 °C; IR 3078, 1685, 1570, 1491, 1422, 1261, 1200, 1085, 972, 819, 655, 513 cm^{-1}; ^{1}H NMR ($CDCl_3$) δ 1.22 (t, J = 7.2 Hz, 3H), 1.23 (t, J = 7.2 Hz, 3H), 3.67 (q, J = 7.2 Hz, 2H), 3.94 (m, 2H), 5.74 (d, J = 7.8 Hz, 1H), 5.95 (d, J = 7.8 Hz, 1H), 7.33 (d, J = 8.5 Hz, 2H), 7.44 (d, J = 8.5 Hz, 2H), 8.14 (d, J = 2.2 Hz, 1H), 8.26 (d, J = 2.2 Hz, 1H) ppm; ^{13}C NMR ($CDCl_3$) δ 11.4, 12.7, 47.4, 50.7, 58.4, 82.6, 85.0, 87.4, 128.7, 128.9, 133.3, 134.2, 134.8, 136.6, 152.8, 158.6, 185.9, 190.8 ppm; EIMS *m/z*: 656.9 ($[M]^+$), 540.8 ($[M-C_5H_{10}NS]^+$), 508.8 ($[M-C_5H_{10}NS_2]^+$), 148.0 ($[C_5H_{10}NS_2]^+$), 116.0 ($[C_5H_{10}NS]^+$); Analysis calcd for $C_{20}H_{18}ClI_2NO_2S_2$: C, 36.52; H, 2.76; N, 2.13; found: C, 36.75; H, 2.88; N, 2.41.

Flavonone 7a; **General procedure**: To a mixture of H_2SO_4/AcOH (1:3 v/v, 3 mL), 3-dithiocarbamic flavonoid **6a** (0.77 g, 2 mmol) was added in several portions. The reaction mixture was heated at 80 °C for 10 min. To the homogeneous mixture $HClO_4$ (70%, 0.5 mL) was added and the crude **7a** was precipitated with water. This was filtered off, dried, and recrystallized from ethanol to give the pure product (0.68 g, 73%) as colorless crystals. Mp 205–206 °C; IR 3011, 1548, 1421, 1202, 1075, 994, 708, 620 cm^{-1}; ^{1}H NMR (DMSO-d_6) δ 3.87 (s, 4H), 3.95 (s, 4H), 6.84 (s, 1H), 7.00-7.60 (m, 9H) ppm; ^{13}C NMR (DMSO-d_6) δ 54.6, 64.2, 75.1, 117.2, 123.1, 124.6,

126.8, 127.1, 127.3, 128.8, 129.1, 129.9, 132.1, 136.9, 151.0, 185.8 ppm; ESIMS *m/z*: 368 (M–ClO_4^-); Analysis calcd for $C_{20}H_{18}ClNO_6S_2$: C, 51.33; H, 3.87; N, 2.99; found: C, 51.53; H, 4.01; N, 3.23.

Flavonone 7b: Yield 60% (0.59 g); mp 177–178 °C; IR 3012, 1560, 1410, 1214, 1070, 997, 714, 619 cm^{-1}; ^{1}H NMR (DMSO-d_6) δ 3.80 (s, 3H), 3.87 (s, 4H), 3.95 (s, 4H), 6.75 (s, 1H), 7.06 (m, 4H), 7.44 (m, 4H) ppm; ^{13}C NMR (DMSO-d_6) δ 54.6, 55.2, 64.2, 74.9, 114.4, 116.1, 117.2, 122.9, 124.5, 126.9, 127.4, 128.8, 129.1, 132.1, 151.0, 160.3, 185.7 ppm; ESIMS *m/z*: 398.08814 (M−ClO_4^-); Analysis calcd for $C_{21}H_{20}ClNO_7S_2$: C, 50.65; H, 4.04; N, 2.81; found: C, 50.86; H, 4.22; N, 3.02.

Flavonone 7c: Yield 76% (0.73 g); mp 155–156 °C; IR 3002, 1561, 1412, 1212, 1074, 999, 710, 618 cm^{-1}; ^{1}H NMR (DMSO-d_6) δ 1.33 (t, *J* = 7.2 Hz, 3H), 1.43 (t, *J* = 7.2 Hz, 3H), 3.80 (s, 3H), 3.92 (m, 4H), 6.72 (s, 1H), 7.10 (m, 4H), 7.44 (m, 4H) ppm; ^{13}C NMR (DMSO-d_6) δ 10.1, 10.3, 53.9, 54.1, 55.2, 75.0, 114.3, 116.2, 117.1, 122.9, 124.7, 127.7, 127.8, 128.7, 129.2, 132.0, 151.0, 160.2, 184.2 ppm; ESIMS *m/z*: 384.10880 (M−ClO_4^-); Analysis calcd for $C_{21}H_{22}ClNO_6S_2$: C, 52.11; H, 4.58; N, 2.89; found: C, 52.26; H, 4.75; N, 3.07.

Flavonone 7d: Yield 71% (0.80 g); mp 250–251 °C; IR 1594, 1466, 1271, 1080, 990, 898, 620 cm^{-1}; ^{1}H NMR (DMSO-d_6) δ 1.35 (t, *J* = 7.2 Hz, 3H), 1.43 (t, *J* = 7.1 Hz, 3H), 3.92 (m, 4H), 6.90 (s, 1H), 7.06 (d, *J* = 8.7 Hz, 1H), 7.57 (m, 5H), 7.77 (d, *J* = 2.3 Hz, 1H) ppm; ^{13}C NMR (DMSO-d_6) δ 10.0, 10.3, 54.1, 54.2, 74.5, 114.2, 118.1, 119.4, 126.8, 127.0, 128.1, 129.1, 129.5, 134.4, 134.6, 135.4, 149.8, 184.3 ppm; ESIMS *m/z*: 467.96793 (M−ClO_4^-); Analysis calcd for $C_{20}H_{18}BrCl_2NO_5S_2$: C, 42.34; H, 3.19; N, 2.46; found: C, 42.51; H, 3.38; N, 2.72.

Flavonone 7e: Yield 64% (0.68 g); mp 244–246 °C; IR 3020, 1559, 1449, 1075, 897, 715, 619 cm^{-1}; ^1H NMR (DMSO-d_6) δ 1.34 (t, J = 7.2 Hz, 3H), 1.43 (t, J = 7.1 Hz, 3H), 3.93 (m, 4H), 6.84 (s, 1H), 7.06 (d, J = 8.7 Hz, 1H), 7.49 (m, 6H), 7.76 (d, J = 2.3 Hz, 1H) ppm; ^{13}C NMR ($CDCl_3$) δ 10.0, 10.3, 54.1, 54.2, 75.3, 114.1, 118.2, 119.3, 126.6, 126.9, 127.5, 128.6, 129.1, 130.0, 134.3, 136.4, 150.1, 184.2 ppm; ESIMS *m/z*: 434.00659 (M−ClO_4^-); Analysis calcd for $C_{20}H_{19}BrClNO_5S_2$: C, 45.08; H, 3.59; N, 2.62; found: C, 45.26; H, 3.74; N, 2.99.

Flavonone 7f: Yield 80% (1.03 g); mp 235–236 °C; IR 1594, 1536, 1466, 1080, 1011, 829, 621 cm^{-1}; ^1H NMR (DMSO-d_6) δ 1.35 (t, J = 7.4 Hz, 3H), 1.43 (t, J = 7.4 Hz, 3H), 3.92 (m, 4H), 7.02 (s, 1H), 7.55 (m, 5H), 7.80 (d, J = 2.2 Hz, 1H), 7.93 (d, J = 2.2 Hz, 1H) ppm; ^{13}C NMR ($CDCl_3$) δ 10.0, 10.3, 54.1, 54.2, 75.0, 111.8, 114.6, 119.0, 126.5, 128.9, 129.1, 129.3, 134.7, 135.0, 136.3, 146.8, 184.4 ppm; ESIMS *m/z*: 511.91912 (M−ClO_4^-); Analysis calcd for $C_{20}H_{18}Br_2ClNO_5S_2$: C, 39.27; H, 2.97; N, 2.29; found: C, 39.38; H, 3.19; N, 2.54.

Flavonone 7g; **General procedure**: To a mixture of P_2O_5/CH_3SO_3H (1:10 m/v, 3 mL), 3-dithiocarbamic flavonoid **6g** (1.25 g, 2 mmol) was added in several portions. The reaction mixture was stirred for 30 min at room temperature. To the homogeneous mixture $HClO_4$ (70%, 0.5 mL) was added and the crude **7g** was precipitated with AcOMe (50 mL). This was filtered off, dried, and recrystallized from ethanol to give the pure product (1.13 g, 80%) as colorless crystals. Mp 238–239 °C; IR 3015, 1554, 1434, 1220, 1075, 1004, 714, 620 cm^{-1}; ^1H NMR (DMSO-d_6) δ 1.34 (t, J = 7.1 Hz, 3H), 1.43 (t, J = 7.1 Hz, 3H), 3.90 (m, 4H), 6.99 (s, 1H), 7.48 (m, 5H), 7.82

(d, J = 1.9 Hz, 1H), 8.12 (d, J = 1.9 Hz, 1H) ppm; ^{13}C NMR (DMSO-d_6) δ 10.0, 10.3, 54.1, 54.2, 75.7, 87.1, 88.1, 118.5, 126.3, 127.2, 128.8, 129.0, 129.9, 132.5, 136.2, 147.3, 150.1, 184.5 ppm; ESIMS *m/z*: 605.89177 ($M-ClO_4^-$); Analysis calcd for $C_{20}H_{18}ClI_2NO_5S_2$: C, 34.03; H, 2.57; N, 1.98; found: C, 34.15; H, 2.71; N, 2.19.

Flavonone 7h: Yield 65% (0.96 g); mp 240–241 °C; IR 1596, 1544, 1427, 1224, 1076, 990, 809, 701, 620 cm^{-1}; ^{1}H NMR (DMSO-d_6) δ 1.36 (t, J = 6.9 Hz, 3H), 1.43 (t, J = 6.9 Hz, 3H), 3.92 (m, 4H), 7.00 (s, 1H), 7.55 (s, 4H), 7.83 (d, J = 1.9 Hz, 1H), 8.13 (d, J = 1.9 Hz, 1H) ppm; ^{13}C NMR (DMSO-d_6) δ 10.0, 10.3, 54.1, 54.2, 74.9, 87.3, 88.1, 118.4, 126.6, 128.3, 129.1, 129.3, 132.6, 134.6, 135.1, 147.3, 149.8, 184.5 ppm; ESIMS *m/z*: 639.85291 ($M-ClO_4^-$); Analysis calcd for $C_{20}H_{17}Cl_2I_2NO_5S_2$: C, 32.45; H, 2.31; N, 1.89; found: C, 32.68; H, 2.52; N, 2.15.

X-Ray structure determinations

Numerical details are presented in Table 1. *Data collection and reduction*: Crystals were mounted in inert oil on glass fibers and transferred to the cold gas stream of the diffractometer (**6a**, **7a**: Oxford Diffraction Xcalibur E with monochromated Mo Kα radiation, λ = 0.71073 Å; **6b**: Oxford Diffraction Nova A with mirror-focused Cu Kα radiation, λ = 1.54184 Å). Absorption corrections were applied on the basis of multi-scans. *Structure refinement*: The structures were refined anisotropically against F^2 (program SHELXL-97 [Sheldrick, G.M. *Acta Cryst. B* **2008**, *64,* 112-122.]). Hydrogen atoms were included by using rigid idealized methyl groups or a riding model.

Crystallographic data have been deposited with the Cambridge Crystallographic Data Centre as supplementary publications no. CCDC-897917 (**6a**), -897918 (**6b**), -897919 (**7a**). Copies of the data can be obtained free of charge from http://www.ccdc.cam.ac.uk/data_request/cif.

Table 1: Crystallographic data for compounds **6a**, **6b**, **7a**.

Compound	**6a**	**6b**	**7a**
Formula	$C_{20}H_{19}NO_3S_2$	$C_{21}H_{21}NO_4S_2$	$C_{44}H_{38}BNO_2S_2$
M_r	385.48	415.51	687.68
Habit	colorless tablet	colorless tablet	colorless prism
Cryst. size (mm)	0.35×0.30×0.15	0.12×0.10×0.04	0.45×0.15×0.10
Crystal system	orthorhombic	monoclinic	Monoclinic
Space group	*Pbca*	*C*2/*c*	*C*2/*c*
Cell constants:			
a (Å)	12.6712(4)	18.1287(5)	30.7002(11)
b (Å)	14.5427(4)	10.0097(3)	10.4557(4)
c (Å)	19.8175(6)	22.1069(6)	22.3121(8)
α (°)	90	90	90
β (°)	90	91.662(3)	100.395(4)
γ (°)	90	90	90
V (Å^3)	3651.8	4009.9	7044.4
Z	8	8	8
D_x (Mg m^{-3})	1.402	1.377	1.297
μ (mm^{-1})	0.3	2.6	0.19
F(000)	1616	1744	2896
T (°C)	−173	−173	−173
Wavelength (Å)	0.71073	1.54184	0.71073
$2\theta_{max}$	57.4	152	60
Refl. measured	72141	58701	125283
Refl. indep.	4688	4163	10252
R_{int}	0.045	0.027	0.052
Parameters	235	254	451
$wR(F^2$, all refl.)	0.086	0.072	0.099
$R(F, >4\sigma(F))$	0.036	0.027	0.040
S	1.06	1.02	1.03
max. Δ/ρ (e Å^{-3})	0.48	0.31	0.52
